# The COVID-19 pandemic: one year later – an occupational perspective

**DOI:** 10.5271/sjweh.3956

**Published:** 2021-04-27

**Affiliations:** Alex Burdorf and Fabio Porru, Department of Public Health, Erasmus Medical Center Rotterdam, Rotterdam, The Netherlands. [email:a.burdorf@erasmusmc.nl]; Reiner Rugulies, National Research Centre for the Working Environment (NFA), Department of Public Health and Department of Psychology, University of Copenhagen, Copenhagen, Denmark. [email: rer@nfa.dk]

About one year ago, we wrote about how the COVID-19 pandemic was unfolding worldwide and started to impact our personal and professional lives (1). Little did we know that, one year later, more than 2.5 million people would have died, with the highest death tolls in Europe, North America, and Latin America (2). Whereas in many countries, life expectancy has increased steadily over the past decades with a couple of months per year, emerging evidence shows that the COVID-19 pandemic will abruptly end this trend in various countries. As one of the most affected countries, life expectancy at birth in the US was down during the first half of 2020 already 1.0 per year compared to 2019 (3). Projections indicate a potential loss in life expectancy of 1.13 years in 2020 for the total US population, resulting in the lowest life expectancy since 2003. The disproportionate burden of COVID-19 mortality is reflected in a staggering loss of 3.1 years in the Latino population and 2.1 years in the Black population (4). It can be expected that,disparities in life expectancy between social and ethnic groups have increased in many countries, demonstrating that COVID-19 has affected different groups differently.

Although deaths attributed to COVID-19 mainly occur among the elderly, often with underlying health conditions, there is scattered evidence that an individual’s type of job may contribute to the risk of becoming infected and, hence, to the mortality pattern in society. One of the first reports has emerged from the UK, where death certificates hold information on occupation. COVID-19-related mortality was highest among men in the lowest skilled occupations, with security guards among the occupation with the highest death rate. Other occupations with increased risks included taxi drivers, chauffeurs, bus drivers, restaurant chefs, and sales and retail assistants. Men and women in social care, including care and home-care workers, had increased mortality, but doctors and nurses in healthcare had death rates similar to the general workforce (5).

This report points to the importance of occupation as a risk factor but also to the availability and use of appropriate personal protection to mitigate the risk of becoming infected. In addition, well-established socio-economic factors of health inequalities intermingled with occupations at risk, demonstrated by the fact that most taxi drivers belonged to the same ethnic group and that taxi drivers had higher mortality rates when residing in London (5). These findings are mirrored in a recent preprint publication from the US state of California, reporting that relative excess mortality was particularly high among food/agriculture, transportation/logistics, facilities, and manufacturing workers. Again, Latino and Black Californian workers were disproportionally affected (6). Hence, working and living circumstances are strongly intertwined, best illustrated by several well-documented outbreaks of COVID-19 in slaughterhouses pointing at working conditions significantly interrelated to housing and transportation arrangements, and precarious work with migrant workers doing the lowest paid jobs (7). A recent large population-based study in Sweden showed that COVID-19-related mortality was influenced by housing conditions (less m^2^ per individual in household; someone of working age in the household), neighbourhood characteristics (higher population density) and educational level (lower education) (8). This raises the question how well we can distinguish the relative contribution of these risk factors to COVID-19, with the added complexity that these risk factors often occur together in vulnerable groups.

There is a lively debate as to which occupations face the highest risks of contracting COVID-19, pointing primarily towards jobs in health and social care dealing with (suspected) COVID-19 patients, and jobs that involve a large number of daily contact with the public or close physical proximity to others. However, clear insight is lacking as access to testing capacity and suitable protective equipment, and organizational and environmental control measures strongly differ across occupations. A large study among more than 2 million users of a COVID-19 symptoms app in the US and the UK showed that frontline healthcare workers reported a 12-fold higher rate of positive COVID-19 tests compared to the general community. After adjustment for the likelihood of receiving a COVID-19 test, by using inverse probability weighting, the increased likelihood of receiving a positive COVID-19 test reduced to a 3.4-fold rate, demonstrating the risk of bias due to access to testing capacity (9). Many studies have been published on infection rates within specific occupational groups, but robust studies at population level across a variety of occupations are needed to investigate the incidence of infection from coronavirus across professions. An illustrative example is the study on SARS-CoV-2 antibody seroprevalence across 18 cities in Iran, which showed rates among healthcare workers comparable to that of supermarket cashiers, pharmacy employees, and hotel staff (10). These findings suggest that the risk resulting from a working environment at higher risk of infection (eg, hospitals) may be mitigated due to effective precautions, while more measures and training may be needed in those settings with a low perception of danger and less trained workers.

From an occupational health perspective, we are not only facing the fatal and non-fatal consequences of COVID-19 but also the indirect effects on mental health. Many authors have reported anecdotal evidence about higher levels of symptoms of anxiety, depression, and post-traumatic disorder among healthcare professionals. These cross-sectional studies are mere indications that workers exposed to COVID-19 patients are psychologically stressed. One of the first longitudinal studies was conducted in Japan, following more than 1000 workers during the two months of the first wave. After adjusting for the covariates, psychological distress (and subscales of fatigue, anxiety, and depression) as well as fear and worry of COVID-19 increased among healthcare workers, whereas psychological stress remained remarkably constant among non-healthcare workers (11).

While the pandemic may broadly affect the mental health of the general population (12), there is a concern that it will particularly adversely affect the mental health of those most vulnerable, ie, those who already had existing mental health problems before the pandemic emerged (13). Keeping and reintegrating individuals with mental health problems in the labor market was already a major public and occupational health challenge prior COVID-­19 (14), and it might become an even bigger challenge in the near future.

Less is still known about the risk to the health of workers who were required to change their regular work practice. The baseline survey of an occupational cohort in the US illustrates the profound impact of the COVID-19 pandemic where 30% of all workers had to work from home, 24% had reduced working hours or income, and 19% were furloughed or placed on leave of absence (15). Working from home may increase flexibility and control, but this may be offset by a non-work-friendly environment (eg, no room to work alone, lack of high-quality internet connection, and no ergonomic working station). A panel study in Germany suggested that particularly women working from home with children were at higher risk of exhaustion, with job autonomy and partner support partly mitigating this effect (16). A small longitudinal study in England found that 72% of workers who changed to remote work experienced increased sedentary behavior, poorer quality of sleep, and more mood disturbances (17). A repeated cross-sectional study, comparing 2016 with 2020, showed more experience and diversity in internet use, but also considerably more ‘techno stress’, defined as individual’s attempts and struggles to deal with constantly evolving ICTs and changing cognitive and social requirements (18).

The long-lasting effects on workers’ health are still unknown. It is a safe bet to accept that work arrangements will not remain the same after the pandemic. We suggest three priorities for the research agenda on COVID-19 and occupational health:

1. Identification of occupations at higher risk for becoming infected and specific work characteristics that contribute to the risks. Such insights will be immensely valuable for preparedness to threats of future pandemics.
2. The impact of COVID-19 on changes in how, where, and when we work, and the consequences for workers’ health, especially mental health. The pandemic has strongly accelerated trends of already existing macroeconomic changes (eg, towards online marketplaces), and there is a need for both occupational health professionals and policy makers to adapt to this acceleration. The traditional workplace may be abandoned for many workers, and new ways must be found on how work will create value for the organization as well as the worker.
3. The impact of the pandemic on social inequalities. This is a great concern as vulnerable groups have been disproportionately affected and their working conditions cannot be isolated from poorer social, economic, and living conditions.

We can only reiterate our previous words: COVID-19 will have both short-term and long-lasting impacts on societies, healthcare systems, workplaces and individuals alike. Occupational health experts are challenged to contribute to a world, especially the world of work, that is a better place after this pandemic.
